# Flow-induced high frequency vascular wall vibrations in an arteriovenous fistula: a specific stimulus for stenosis development?

**DOI:** 10.1007/s13246-023-01355-z

**Published:** 2023-12-29

**Authors:** Michela Bozzetto, Andrea Remuzzi, Kristian Valen-Sendstad

**Affiliations:** 1 Bioengineering Department, Istituto di Ricerche Farmacologiche Mario Negri IRCCS, Ranica, BG Italy; 2 Department of Management Information and Production Engineering, University of Bergamo, Via G.B. Marconi 5, Dalmine, BG Italy; 3 Department of Computational Physiology, Simula Research Laboratory, Oslo, Norway

**Keywords:** Arteriovenous fistula, Transitional flow, Vascular wall vibrations, Mechanobiology

## Abstract

Hemodialysis is the lifeline for nearly three million end stage renal disease patients worldwide. Native arteriovenous fistula (AVF) is the preferred vascular access, but 40% fail within 1 year. We recently demonstrated that AVFs harbour transitional flows and the goal of the present study was to investigate whether the associated high-frequency pressure fluctuations could promote vibrations within the vascular wall. We acquired MRI images and flow rates immediately after surgery in one patient and generated a 3D patient-specific model. High-fidelity fluid structure interaction simulations revealed the presence of wall vibrations in distinct frequency bands up to 200 Hz and amplitude of 200 μm. A sensitivity analysis to assess the impact of flow rates, and vascular wall stiffness and thickness, changes that typically occur during AVF maturation, confirmed the robustness of the results. Interestingly, the vibrations were always predominant at the anastomosis floor and on the inner venous side, which correlates with typical stenotic regions. As studies seeking to correlate aberrant stresses and vascular remodelling have been largely inconclusive, the focal colocalization between vibrations and stenosis may suggest an unknown mechanobiological process between high-frequency mechanical stresses within the vascular wall and adverse vascular remodelling.

## Introduction

End stage renal disease (ESRD) is a leading cause of death and disability worldwide [1], posing an economic burden on the healthcare systems. The number of patients exceeded 2.5 million in 2015 and is expected to exceed more than 5.4 million by 2030 [2]. The disease cannot be cured and the lifeline for most of the patients is hemodialysis treatment every second day through a surgically created arteriovenous fistula (AVF). Despite AVFs having better survival and less complications compared to synthetic grafts and central venous catheters, 40% of them fail within 1 year after surgery [3] because of an adverse inward stenotic remodelling. As a consequence, patients undergo frequent hospitalisation and emergency re-interventions. Management of AVF patients remains a major clinical dilemma as there are still no effective strategies to predict or prevent AVF stenosis, mainly due to the limited understanding of the underlying pathogenic mechanisms.

Over the past decades it has become evident that local hemodynamic conditions contribute to wall adaptation and remodelling in healthy vessels [4], and to vascular pathogenesis [5]. The stresses at the vascular wall cannot be measured directly, but routinely available medical images have been used as input to computational fluid dynamic (CFD) simulations in the investigation of various patient-specific vascular pathologies [6]. More specifically in AVFs, the leading hypothesis is that the surgical procedure of AVF creation alters the hemodynamic stresses with subsequent vascular remodelling to restore initial physiological hemodynamic conditions [7]. Particularly for the AVF, the wall thickness increases and the vessel dilates to re-establish basal levels of intramural tensile stress and wall shear stress, which is a naturally occurring and desired maturation process that also causes increased flow rates. This also highlights that the remodelling is purely mechanically driven in the AVF. However, not all vessels mature and an aberrant inwards stenotic remodelling can occur both immediately after surgery and over a longer time period. Nevertheless, after decades of flow studies, the correlation between local aberrant stresses at the vascular wall with stenosis formation appears both inconsistent and inconclusive. Modelling of vascular diseases can be the solution to certain clinical problems, but only a plausible model can advance the knowledge on the pathogenic process.

Nevertheless, from a biological point of view, there are some logical fallacies that might have been overlooked. The vast majority of metrics and indices proposed and computed in the literature are inspired by correlations between flow, gene and protein expressions from experiments performed on intact endothelium [4]. There is admittedly no doubt that there are distinct gene and protein expressions associated with different flow phenotypes. However, AVFs are artificially created and damage to the endothelium during surgery is unavoidable. Since re-endothelialization may take time to occur, the wall might be completely insensitive to stresses acting at the luminal surface. Secondly, neointimal hyperplasia is the first manifestation of disease and the wall is in a pathological state by definition [8]. It is questionable how the vascular wall would respond to potential stimulus, if the endothelial cells had not been damaged during surgery in the first place. However, there is still a focalised remodelling which means that there might be a different stimulus the wall can sense and our hypothesis is that focal wall dysfunction may not only depend on stresses at the endothelial cells. There are therefore strong indications that AVF remodelling and eventual failure is not only a flow problem of the luminal surface, but may involve a mechanobiological mechanism within the vessel wall itself. Previous studies successfully conducted fluid-structure interaction (FSI) analyses and adequately addressed their particular research inquiries [9, 10]. However, it’s worth noting that in each of these investigations, the simulations were conducted with the underlying assumption of laminar flow. In contrast, we previously demonstrated that transitional flow, characterised by high-frequency velocity and pressure fluctuations, is present in the venous segment of the AVF [11], and others have later reported turbulence in the same vascular region [12]. Therefore, the primary goal of the current study was to investigate whether these high frequency pressure fluctuations could give rise to wall vibrations in a patient-specific AVF, with a secondary goal to evaluate the impact of the flow rates and also vascular wall thickness and stiffness, which typically vary during AVF maturation.

## Materials and methods

### Patient-specific three-dimensional model generation

This study received approval by the Bergamo Ethics Committee (Reference number NCT04141852). The patient authorised the use of his clinical and imaging data for research purposes. A contrast-free MRI was acquired in a 72-year-old male 3 days after the creation of a native distal radio-cephalic side-to-end AVF. We acquired the CUBE sequence, previously optimised for AVF imaging [13] on a 1.5T scanner (GE, Optima 450w GEM) using a flexible 16-channel phased array medium coil, covering a region of approximately 3 and 5 cm above and below the anastomosis, respectively. Image segmentation was performed using the Vascular Modelling ToolKit [14]. Straight cylindrical flow extensions of five diameters were added to the vessels to ensure fully developed blood velocity at the inlets.

### The computational domain

**Fig. 1:**
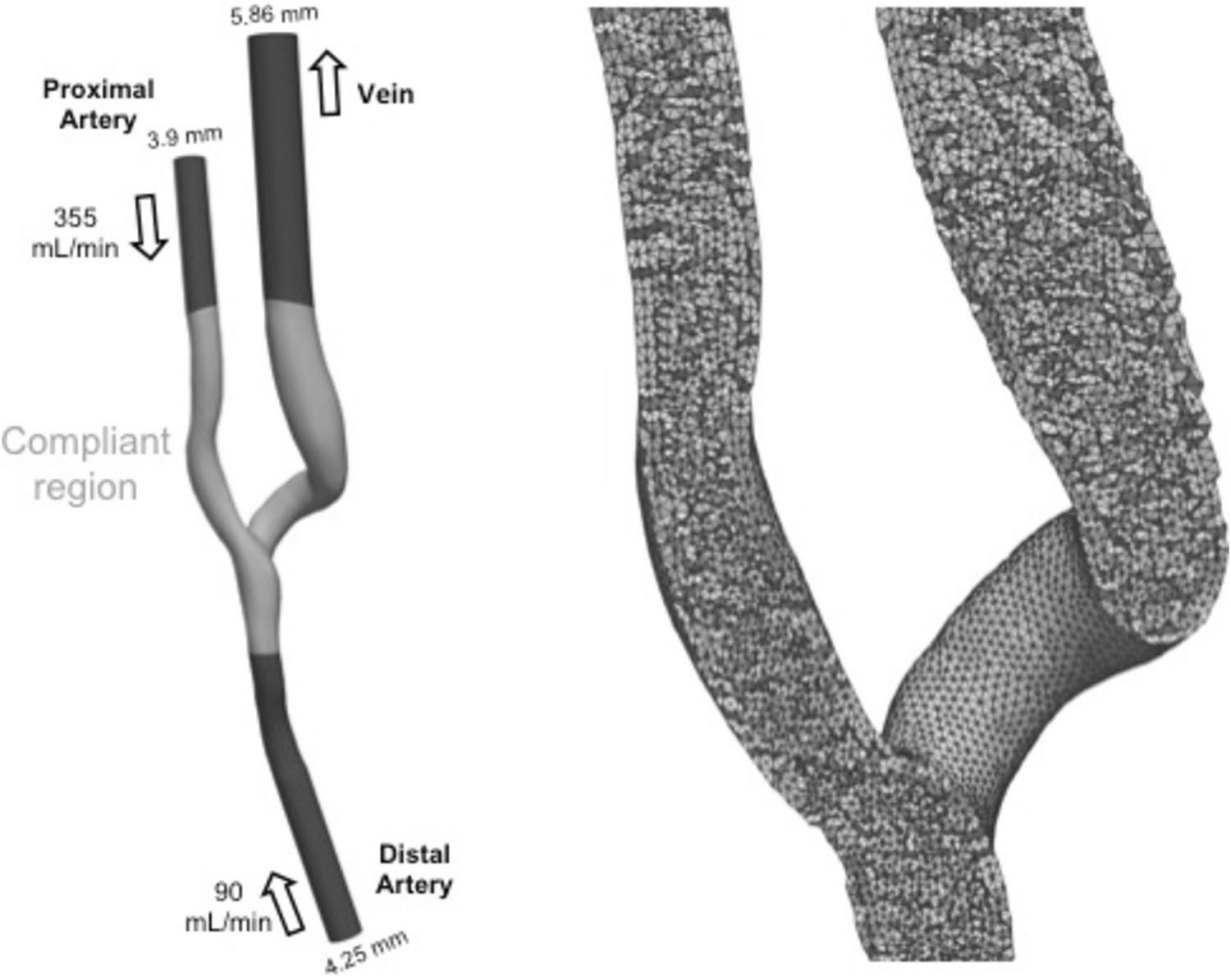
Representation of the 3D AVF model (left) with dimensions, flow rates, flow extensions and the rigid versus compliant domains, respectively. The computational mesh is shown on the right

The computational domain was discretized using a mesh consisting of 400 thousand tetrahedral elements, with a mean nodal edge length of 0.256 mm. An external layer with a constant thickness of 0.3 mm was added to model the vascular wall [15] (see Fig. 1). The extremities of the model were assumed to be rigid and fixed in space while the rest of the domain was compliant [16]. The vascular walls were modelled as St. Venant-Kirchhoff hyperelastic material [9, 17], with a Young’s modulus of 2 MPa [18], a Poisson ratio of 0.45, and a density of 10^3^ kg/m^3^.

### Numerical simulations

High-fidelity fluid structure interaction (FSI) simulations were performed using the open-source solver turtleFSI [19]. turtleFSI is a validated, fully coupled, monolithic and space/time-centred solver, which provides a 2nd order accurate solution in time. Quadratic Taylor-Hood elements (P2-P1) for the velocity-pressure field and quadratic P2 elements for the solid deformation were used, which are P + 1 accurate (L2). The latter is equivalent to at least 3.2 million linear elements and the effective node spacing becomes 0.128 mm. An efficient second-order Laplace equation was used for mesh lifting operations, which is most suitable for small deformations. The approximate computing time was 20 h for one physical second on a compute node with 40 Intel Xeon-Gold 6138 2.0 GHz cores.

Blood was modelled as a Newtonian fluid with density and viscosity of 1.025 g/cm^3^ and 3.5^−6^ m^2^/s, respectively. We initialised the simulations by first solving the Stokes flow to avoid artificial oscillations in the initial conditions. The 3D transient problem advanced in time with a timestep of 0.2 ms (equivalent to 5000 timesteps per second) to adequately resolve potential high-frequency velocity and pressure fluctuations. At the inlets we prescribed fully developed parabolic velocity profiles whose magnitude gradually increased over the time interval 0–0.1 s, and thereafter kept constant. The velocity magnitude was obtained from Doppler Ultrasound measurements, and the peak systolic flow rates (Q_PS_) corresponded to 355 mL/min and 90 mL/min in the proximal and distal arteries, respectively. We used a Neumann boundary condition at the outer solid wall, and to mimic the stress equilibrium at the point of image acquisition, i.e., to balance the pressure and intramural tensile stress, the (zero-pressure) mesh was first shrunk by 5% and gradually pressurised up to 10 mmHg to recover the initial patient-specific geometry. Pressurisation was performed after the velocity ramp up, over the time period of 0.05 to 0.2 s, to avoid flow reversal at the vein due to the increased volume of the computational domain.

Being our best estimate for simulation parameters, we refer to the computational setup described above as our default one. However, we also performed additional simulations by varying the blood flow rate, imposing end-diastolic (190 mL/min) and cycle-averaged (270 mL/min) flow rate in the proximal artery (keeping the measured flow rate of 90 mL/min in the distal artery constant), to evaluate the potential of wall vibrations to persist throughout the cardiac cycle. Moreover, using the default flow rates, we then investigated the effect of different vessel wall thickness (0.15 and 0.6 mm) and wall stiffness (4 MPa and 10 MPa), which typically change during the maturation process in AVFs.

### Post-processing of the results

The post-processing focused on the velocity of the fluid and the displacement of the solid domain. For the fluid, we computed the turbulent kinetic energy (TKE) as

$$TKE = \rho ~\frac{1}{2}~\left( {\underline{{\left( {u^{\prime}} \right)^{2} }} + \underline{{\left( {v^{\prime}} \right)^{2} }} + \underline{{\left( {w^{\prime}} \right)^{2} }} } \right)$$

where $u^{\prime }, v^{\prime }, w^{\prime }$are the fluctuating components of the fluid velocity, obtained by subtracting the cycle-average component from the instantaneous component, i.e. $u^{\prime} = u_{i} - \underline{u}$, $v^{\prime} = v_{i} - \underline{v}$ and, $w^{\prime} = w_{i} - \underline{w}$ [20].

For the solid, we computed the time averaged displacement in the entire domain, after the careful increase of flow and pressure (after 0.2 s) to minimise transient effects associated with artificial initial conditions. For the solid, we also computed the spectrograms [21], which were designed to mimic clinically obtained Doppler ultrasound spectrograms. This was done on the venous segment, from the anastomosis up to − 2 diameters from the start of the rigid cylindrical outflows, on the time interval 0.1–1 s. Briefly, the power spectral density (PSD) was calculated based on a Short-Time Fourier transform of the instantaneous deformation averaged over each node in the mesh. A logarithmic scale was applied to the time-averaged PSD converting the values to decibel (dB) to emphasise the higher frequency spectral content.

## Results

**Fig. 2:**
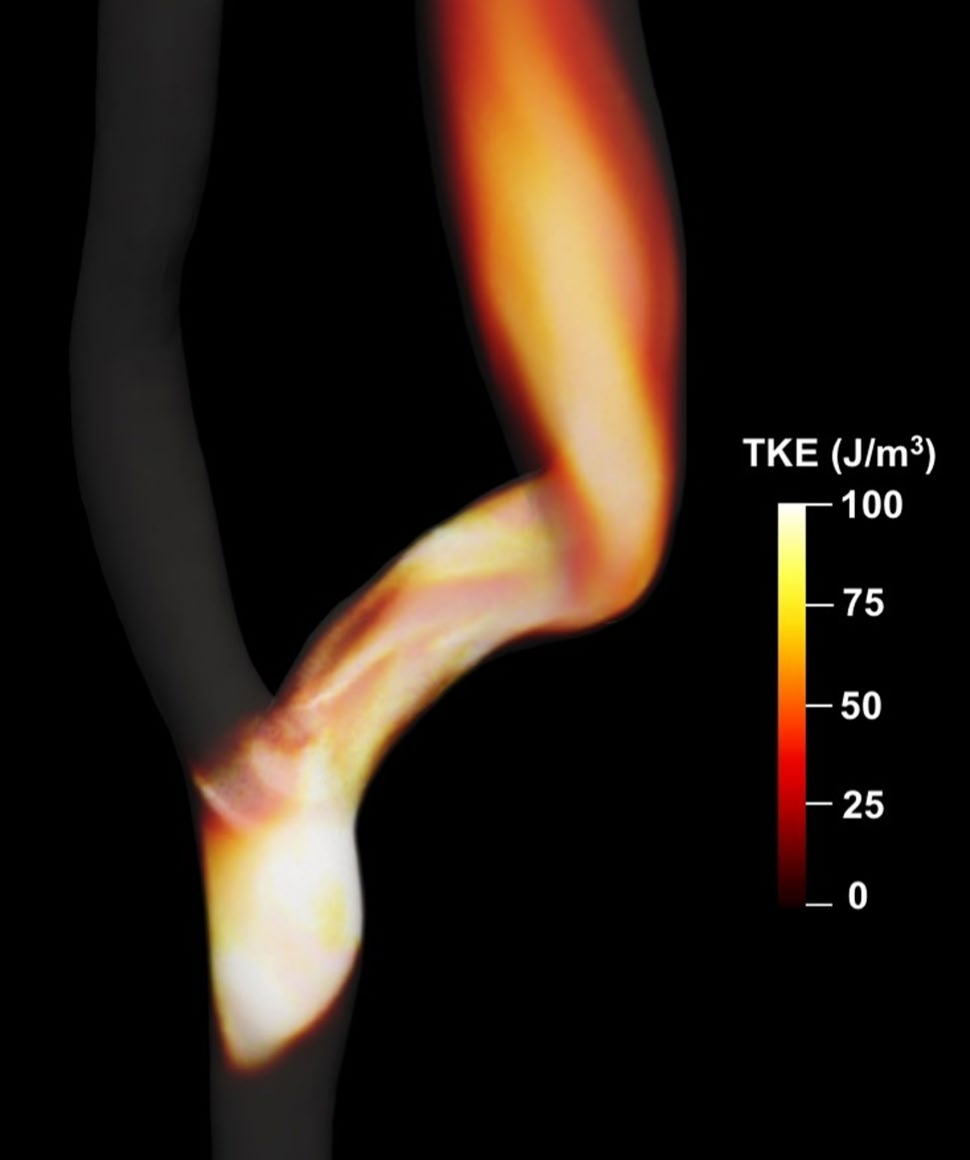
Volumetric rendering of the Turbulent Kinetic Energy (TKE) for the peak systolic blood flow simulation

Focusing first on qualitative results from the default simulation, Fig. 2 shows the volumetric rendering of the TKE. We can observe the absence of TKE in the proximal and distal artery, which is indicative of a laminar flow. In contrast, point values of TKE up to 400 J/m3 are present at the anastomosis and in the juxta-anastomotic vein, while lower TKE values up to 100 J/m3 can be observed in the distal venous segment. The instantaneous flow features are consistent with our previous reports where we first described transitional flow features, see [11].

**Fig. 3:**
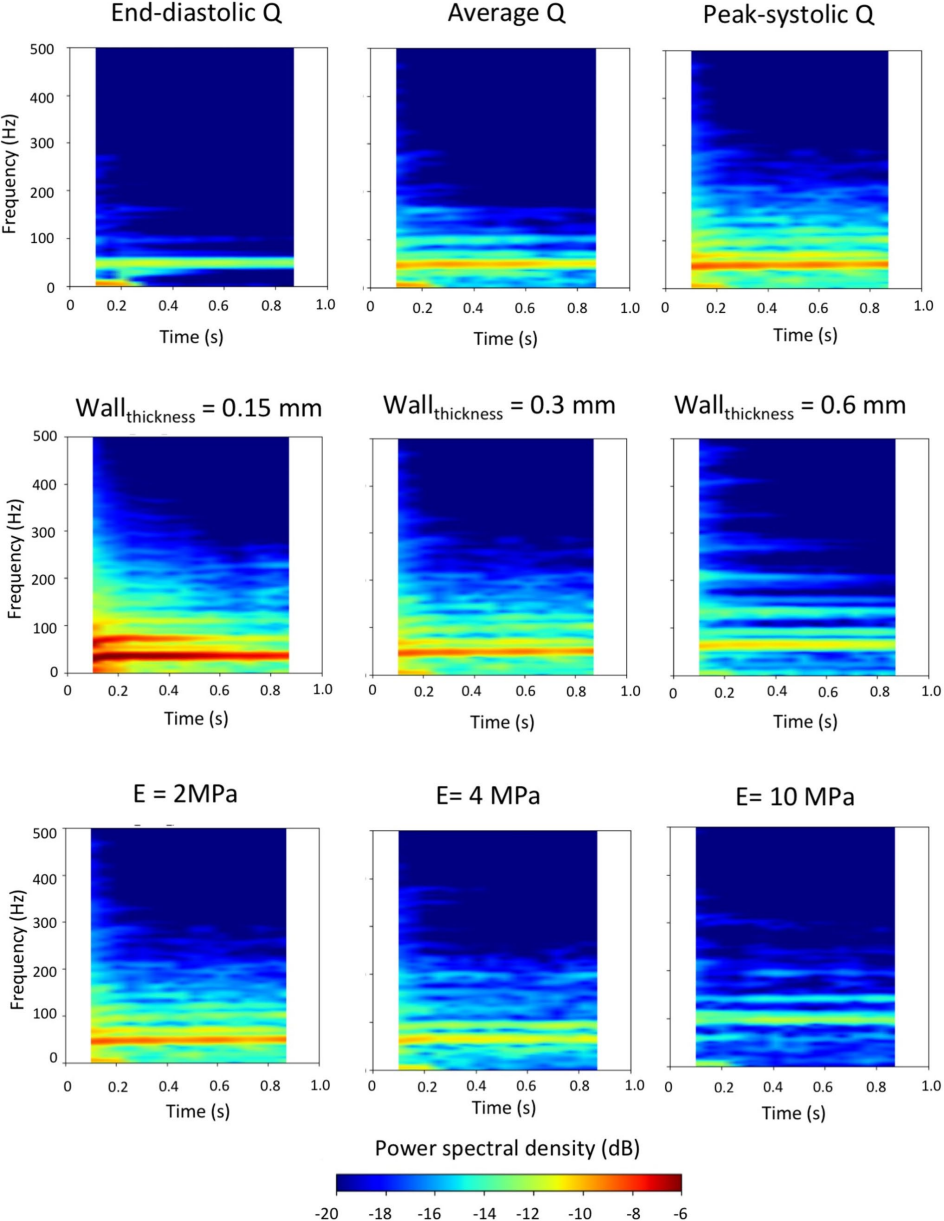
Spectrogram representation of the frequency content of the displacement in the vein for the simulations performed in the parametric study. Abbreviations: Q, blood flow volume; E, Young’s Modulus

Focusing on frequency content in the vein only, more quantitative results are shown in Fig. 3, which demonstrates the effect of varying the blood flow rate (top), wall thickness (middle), and wall stiffness (bottom). For the end-diastolic flow rates, we can see that there is a weak vibration at approximately 60 Hz. Increasing the flow rates led to distinct bands at 60 and 100 Hz for both cyclic average and peak systolic flow rates. Moreover, for the latter, we observe an additional distinct band at 120 Hz and a broadband spectral content between 150 and 220 Hz with amplitude between − 16 and − 18 dB. Varying the wall thickness of the vessels also caused changes both in the amplitude and magnitude of the wall vibrations. In particular, the lowest thickness shows two high amplitude bands of − 6 dB centred around 40 and 70 Hz. Increasing the thickness, these bands decrease in amplitude, up to − 10 dB with vessel thickness of 0.6 mm. More specifically, while a broadband spectrum between 150 and 220 Hz with amplitude between − 16 and − 18 dB was present for vessel thickness of 0.15 and 0.3, the vibrations were significantly reduced, as confirmed by the spectrogram for the 0.6 mm thick vessel. In this case, besides the common feature of a high-energy band slightly increased in frequency up to around 60 Hz, distinct low amplitude narrow bands are present only up to 130 Hz. Increasing the stiffness of the vessel wall resulted in a decrease of the amplitude of vibrations, as shown in the simulation with the highest Young’s modulus, where only two distinct bands were displayed at around 100 and 120 Hz.

**Fig. 4:**
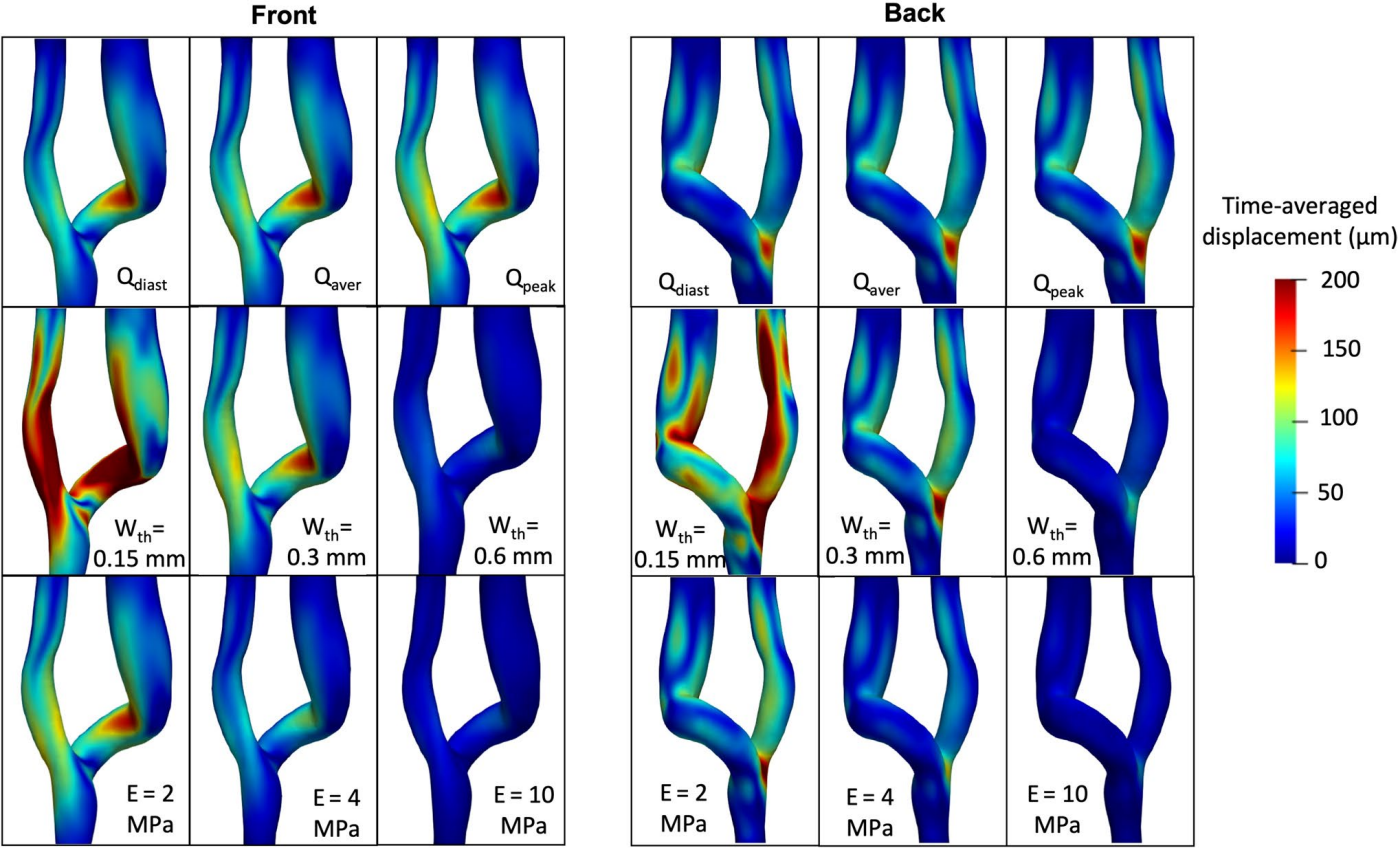
Surface maps of the time-averaged displacement of the wall, front and back, respectively. *Q* blood flow volume, *E* Young’s Modulus; diast, end-diastolic; aver, average; peak, peak systolic; Wth, wall thickness

The corresponding time-averaged displacement in the entire model is shown in Fig. 4. Note that the default simulation results are repeated on the diagonal (lower left to top right) for comparison. Focusing first on the top row, the highest values of 150–200 μm were consistently found at the inner curvature of the vein and at the anastomosis floor. It appears evident that the AVF vibrates regardless of the blood flow rate. In contrast, varying the wall thickness has a considerable effect on the vibration magnitude, as shown in the middle row of Fig. 4. With wall thickness of 0.15 mm (left), both the artery and the vein show high displacement values, which are significantly decreased, but still present with a thickness of 0.6 mm (right). The same behaviour can be observed by varying the stiffness of the vessels (bottom row), with high magnitude vibrations for Young’s modulus of 2 and 4 MPa (left and middle) and markedly reduced magnitude vibrations with a stiffness of 10 MPa (right), as also confirmed in Fig. 3.

## Discussion

We have shown for the first time that transitional flows with high-frequency pressure fluctuations caused the venous wall in an AVF to vibrate at frequencies up to hundreds of Hz. Our results are consistent with clinical reports of bruits, and thrills that can be felt on the skin [22]. We argue that the results are fully plausible and potentially widespread as many AVFs harbour transitional flows. In the following, we will discuss potential implications, address the mechanobiological relevance, and contextualise the results.

**Fig. 5:**
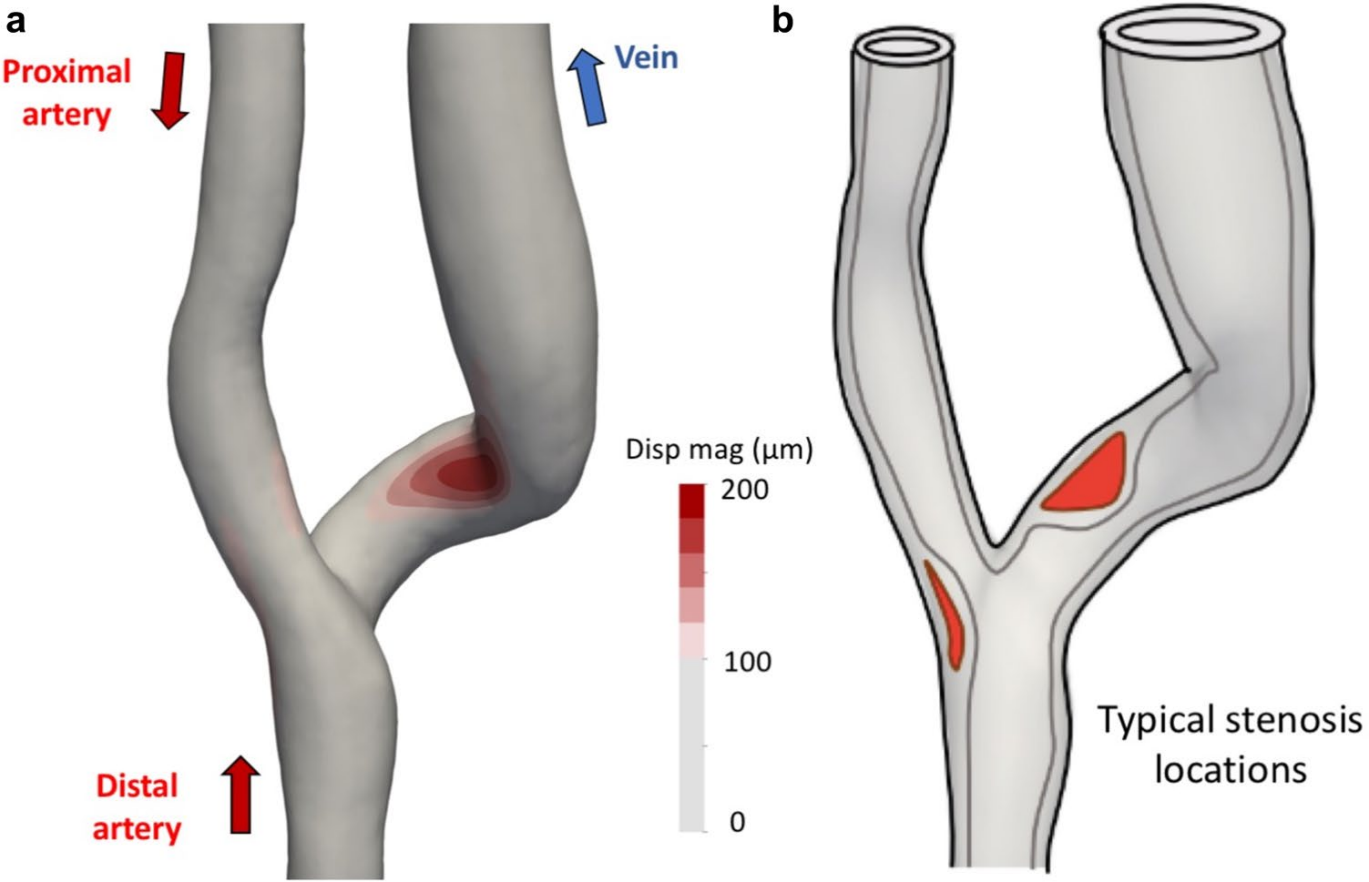
Correlation between vibrations and regions of stenosis formation. **a** time averaged deformation magnitude, **b** region of typical stenosis formation

First of all, except for flows within the heart itself [23] and the aorta [24], “turbulence” in the vascular system is considered relatively rare and normally linked to pathological states. Setting the observed transitional flows in a systemic perspective, there are a few other vascular territories we can compare against. E.g., Dyverfeld et al. [25] reported TKE levels of up to approximately 1000 J/m^3^ in an aortic coarctation, whereas Andersson et al. [26] reported slightly less than 500 J/m^3^. Setting aside the minor differences in methodology and MRI derived calculations, the TKE magnitude observed here is lower, which seems reasonable given the smaller sized vessels. However, the TKE values are comparable to approximately 200 J/m^3^ as previously reported in aneurysms [27, 28] that are vessels of comparable size and flow rates. These computational studies typically investigated the correlation between TKE intensity and disease, hypothesising that flow fluctuations affected the endothelium with subsequent remodelling of the wall. However, those studies were performed under the assumption of rigid walls, and consequently didn’t address potential wall vibrations. The latter highlights the novelty of the current study, and more precisely that the dynamical mechanical stresses, or vibrations, could be a mechanobiological stimulus acting on the smooth muscle cells (SMC) with relevance for vascular remodelling. E.g., more specifically in the AVF, the vibrations were most predominant at the anastomosis heel and at the inner curvature of the vein, where more pronounced vascular remodelling is known to occur after intervention. To contextualise the potential importance of vibrations, Fig. 5, panel (a) shows the time averaged deformation in our model, and inspired and adopted by [8, 29], panel (b) illustrates regions of typical stenosis formation in the AVF. It is evident that the region of the vein with maximum vibration shows a correlation with regions where stenosis typically occurs. Therefore, vibrations in this case seem to be both sensitive and specific to where stenoses develop generally [29]. That being said, there were also low magnitude vibrations upstream of the anastomosis heel that coincide roughly with common sites of stenoses although less frequently. Also, it must be mentioned that there are low magnitude vibrations located on the arterial side, where the vascular walls are typically thicker physiologically and have different mechanical properties as compared to the vein.

### Mechanobiological relevance

Suggesting that there might be a correlation between vibrations and focalized vascular remodelling might seem misplaced, but clinical studies have provided overall evidence at least from the 1960s and sporadically onwards. In 1963 Roachet al. [30] made the observation that post stenotic dilatations are common in a wide range of diseases, and that there is always a bruit present. The authors at the time investigated the link between arterial stenoses, turbulence, bruits and wall vibrations rigorously both in vitro and in vivo. One of Roach’s conclusions was that ‘*the […] arteries of adult dogs developed poststenotic dilatation only, but always, if the stenosis created experimentally produced distal turbulence as defined by the presence of a thrill and bruit*’. The dilatations occurred in nearly any species and vessel, which remodelled actively for up to ~ 10 days before stabilising. This concept was further investigated experimentally by Foreman et al. in 1970 [31], who reported a few years later that flow in isolated arteries with an artificial stenosis ‘*excites the artery wall to vibrate over a wide range of frequencies within which are discrete frequencies that coincide with the resonant frequencies of the artery wall.*’ The latter strongly resembles our observations in the AVF.

However, the previous clinical studies investigating the role of vibrations on vascular remodelling were focused on the arterial side, while Fillinger et al. [32] in 1990 performed the first, and maybe the most authoritative, investigation on the role of vibrations on the venous side. First of all, Fillinger clearly demonstrated the existence of vessel wall vibrations in a canine loop graft using Colour Doppler imaging and even quantified perivascular tissue vibration. More importantly, Fillinger demonstrated clear correlations between vibrations and intimal hyperplasia. Unfortunately, neither Roach nor Fillinger was able to prove whether the main stimulus was through shear stress on the endothelium or through mechanical stress within the vascular walls, as the two (abnormal) stresses coincide in space and time. However, concomitantly, there were two influential papers, first demonstrating that endothelial cells respond to wall shear stress [33], and later to turbulent wall shear stress [34], which apparently shifted research efforts towards stresses acting upon the endothelium, and not the SMCs within the vascular wall itself.

That being said, also recent excellent review papers do indeed mention that ‘*SMCs are highly sensitive to changes in applied loads*’ [35], but the reported frequencies are typically below one Hz. To our knowledge, the only research investigating the effects of higher frequencies on SMCs was performed by Bittle in 1993 [36]. Setting circulatory factors aside, Bittle reasoned that atherogenesis couldn’t be fully explained by adverse or abnormal shear stress in isolation. It was argued that the vibrations caused an acceleration of .1 g that shook the microanatomy of the SMCs that led to an intra cellular collision similar to organ damage that is caused by “relative or different motion of the viscera and the supporting skeletal structure”. Bittle therefore exposed SMCs to vibration and found that small vibration amplitudes up to 45 Hz had no effect on the cells, but cells ‘*subjected to vibration levels of 12 micrometres 45 Hz grew at a significantly higher rate than did cells grown in static conditions.*’ Bittle investigated SMC proliferation with atherosclerosis as the motivating factor, but the experiment nonetheless demonstrated that SMCs are sensitive to such vibrations. Later, the effects of dynamic variations of mechanical stress on SMC biology have been almost neglected in the vascular biology literature. Again, the idea that vibrations may affect cellular remodelling might seem misplaced, but is in fact an active topic of research in e.g. both bone disorders (osteogenesis) [37, 38] and cancer treatment [38].

### CFD and FSI studies

We are admittedly not the first to address potential determinants of stenosis formation computationally, and there are noteworthy rigid wall CFD simulation studies. Lee et al. [39] already in 2005 investigated the correlation between pressure fluctuations from direct numerical simulations and vein wall vibrations recorded with a laser Doppler vibrometer in a canine animal arteriovenous graft. They did not find a good correlation and listed a number of possible reasons among which the rigid wall assumption was mentioned. More importantly, they did not sacrifice the animal and perform histological analyses and couldn’t therefore speculate on any biological mechanisms. On the contrary, Krishnamoorthy et al. [40] reported a correlation between anatomical configuration, wall shear stress profiles, and intima-media thickening in a porcine model of AVF stenosis. In the following study [41] they investigated the influence of WSS patterns over several days and concluded that the increase in WSS magnitude over time was associated with inward remodeling and subsequent stenosis formation. However, their main focus was on the discrimination between curved and straight AVF configurations and less attention was put on a deep characterization of hemodynamic variations over time. About the same time, Ene-Iordache & Remuzzi performed CFD simulations in idealised models [42] and hypothesised about a possible correlation between disturbed flow and stenosis formation in AVFs, highlighting the need for longitudinal studies. He et al. [43] were the first to develop a pipeline from contrast-free MRI to CFD using longitudinal data, and assessed hemodynamics and remodelling in 1 patient at 4, 5, and 7 months. Their methodology was rigorous and well described but they focused primarily on the feasibility of the technique and hypothesised about potential future clinical applications. A similar pipeline was applied by Sigovan at al. [44], who studied 3 patients preoperatively and at 1 and 3 months, focusing again on the feasibility of the technique. Regarding hemodynamic stresses, neither He et al. nor Sigovan et al. found any correlation with stenosis formation and could not provide substantial mechanobiological insights. In conclusion, the results in the literature seem inconsistent and provide only incremental insights into the pathogenesis of AVF failure.

There are also a handful of FSI studies implicitly or explicitly assuming laminar flow and mainly investigating the effects of pulsation (~ 1 Hz) on WSS. Specifically, McGah et al. [9] investigated a single model obtained from 3D ultrasound scans, and compared results against their previous rigid wall simulations studies, having measurement of wall motion to validate against. In their relatively sophisticated model, they found WSS in the range of 10–15 Pa, which was 50% lower than CFD derived results, but since both modelling approaches revealed high WSS, they concluded the CFD was acceptable. The same year, an equally complex AVF model was published by Decorato et al. [45]. They included a non-Newtonian model, but also wall heterogeneity by varying the mechanical properties of the vascular walls. Their study included validation against in-vitro experiments and compared Newtonian and non-Newtonian rheology and heterogeneous vs. homogeneous wall properties. Their main finding was that different modelling assumptions resulted in WSS varying by 20%. De Villiers et al. [10] followed the same ideas and investigated a single model that they validated against MRI measurements. Their rather mathematical focus, which indeed is excellent, somehow overshadowed the study, and their discussion was focused on technical aspects. In strong contrast, Pike et al. [17] focused on more mechanobiological aspects and *‘hypothesize[d] that NOS3 promotes AVF maturation by regulating local vascular mechanics following AVF creation’* which was varied in murine models. In their paper, FSI seemed only to be performed to infer WSS in the AVF models, again where wall motions were prescribed based on medical images. In conclusion, in all of these studies the authors have performed FSI and answered their specific research questions adequately, but all simulations were performed under the assumption of laminar flow.

### Strengths and limitations

Setting aside general limitations of patient-specific modelling of the cardiovascular system related to potential errors during segmentation [46] and the assumption of Newtonian rheology [47–49], there are a couple of specific limitations associated with the current study. First of all, we have not presented a formal mesh convergence study, but simulations performed on meshes ranging from 100 to 800k with time steps between 5 and 15k showed qualitative similar results. However, blind focus should not be on the mesh size alone compared against low order methods, as we are using Taylor-Hood elements for the fluid dynamics and quadratic elements for the deformation with third order accuracy (L2), that also capture boundary layers. Additionally, the mesh size and time step size used in this study corresponds to the ‘fine’ mesh resolution previously shown to be (more than) adequate to resolve such transitional flows [50, 51]. Secondly, we have used a steady state inflow velocity which is clearly unphysiological and can be easily improved using a pulsatile waveform in the next study, but the use of constant flow rate is simply a pragmatic choice that allows us in the simplest possible way to reveal whether the vascular wall may vibrate. We obtained an easier isolation of flow instabilities and associated wall vibrations, and we showed that they are triggered by non-physiological elevation of the blood flow rate in the AVF venous vessel morphology. That being said, we also investigated the measured patient-specific cycle-averaged and end diastolic flow rates, and the wall vibrated regardless. If anything, the use of constant flow rates would underestimate flow instabilities, and presumably vibrations, that typically intensify during peak deceleration due to adverse pressure gradients [52]. This doesn’t seem completely unreasonable given that the clinically reported bruits acquired through auscultation are normally at the order of 500 Hz, compared to the maximum of 300 Hz in the current study. In addition to that, the patient-specific data used in the current study was from a 3-day old AVF, that had not yet matured and the flow rates were ‘only’ 445 mL/min, which is 155 ml/min lower than what is considered a well–functioning AVF (AVF’s Rule of sixes).

Therefore, whether the observed vibration modes in Fig. 3 are physical or physiological can be debated, but in the case of the former, it simply shows that the model represents the behaviour of ‘any’ mechanically coupled system which further validates the methodology. Physiologically, the predicted frequency magnitude and amplitude can naturally also be affected by the perivascular environment, which is not incorporated in the current model. Turning to experiments, e.g., Foreman et al. [30] reported that *‘[c]alculations based on the specifications of the transducer suggest that the vibrations are of the order of 0.01 mm peak to peak’*, which is comparable to the results of Balasso et al. [53] who used a silicone phantom to study vibrations in an aneurysm. These two laboratory experiments suggest that the vibrations in our computational study are an order of magnitude too large, which can be attributed to the lack of external tissue in our computational model, and potentially sensitivities to the region of the compliant domain. As such, our mathematical model would mimic an experimental laboratory setup, but physiologically, the AVF is nominally close to the skin, which should have a minimally damping effect. These phenomena are nevertheless confirmed in preclinical studies where bruits (and vibrations) have been observed “deep” (1,5 cm) within the animal in the femoral and carotid arteries [30]. The perivascular tissue vibration was also confirmed by Fillinger et al. [32] who *‘quantitated [vibrations] by the distance required for Doppler signal attenuation’*. Finally, a recent study conducted in the carotid bifurcation has shown by a Doppler laser vibrometer that in presence of turbulent-like flow the wall of this vessel vibrates at amplitude and frequencies that are comparable to the ones found in this study [54].

Finally, we also assumed a constant thickness of the vascular walls. However, the vein is normally in a low-pressure regime and much thinner than the artery before maturation. Also, the stiffness of the vasculature (Young´s modulus) was unknown and estimated based on healthy vascular walls. Setting aside the impact of varying Young’s modulus, flow rates, and wall thickness, which confirms that these factors are sensitivity issues, we would argue that the overall physiological phenomenon is fully plausible. Moreover, vibrations are determined by the morphology and presence of the pressure fluctuations in the flow, i.e., the local Reynolds number, which is uniquely determined by the AVF morphology and the flow rates. In our case, the generation of the morphology from patient-specific medical images and derivation of flow rate from velocity measurements by Doppler Ultrasound definitely minimise the study limitations. Although our observations are limited to one patient only, they are based on patient-specific data and are consistent with clinical reports on thrills that can be felt on the skin and that give rise to audible bruits, which change over time when stenosis develops. Indeed, AVF sound analysis may predict the development of stenosis and could ultimately be directly applicable in routine clinical practice.

## Conclusion

We have shown for the first time that transitional flows with high-frequency pressure fluctuations caused the walls in an AVF to vibrate at frequencies up to hundreds of Hz. Moreover, the phenomenon could also be potentially widespread as many AVFs harbour transitional flows. There is no doubt that WSS is important in normal physiological vascular remodelling, but the current results suggest that wall vibrations, or more precisely dynamic mechanical stress, may be involved in vascular remodelling and that vibrations could directly affect SMC mechanobiology.
